# The effects of mango leaf extract during adolescence and adulthood in a rat model of schizophrenia

**DOI:** 10.3389/fphar.2022.886514

**Published:** 2022-07-26

**Authors:** Jose Antonio Garcia-Partida, Sonia Torres-Sanchez, Karina MacDowell, Maria Teresa Fernández-Ponce, Lourdes Casas, Casimiro Mantell, María Luisa Soto-Montenegro, Diego Romero-Miguel, Nicolás Lamanna-Rama, Juan Carlos Leza, Manuel Desco, Esther Berrocoso

**Affiliations:** ^1^ Neuropsychopharmacology and Psychobiology Research Group, Department of Neuroscience, University of Cádiz, Cádiz, Spain; ^2^ Instituto de Investigación e Innovación en Ciencias Biomédicas de Cádiz, INiBICA, Hospital Universitario Puerta del Mar, Cádiz, Spain; ^3^ Neuropsychopharmacology and Psychobiology Research Group, Psychobiology Area, Department of Psychology, University of Cádiz, Cádiz, Spain; ^4^ Ciber of Mental Health (CIBERSAM), ISCIII, Madrid, Spain; ^5^ Department of Pharmacology and Toxicology, Faculty of Medicine, Universidad Complutense de Madrid (UCM), Health Research Institute Hospital 12 de Octubre (imas12), Institute of Research in Neurochemistry IUIN-UCM, Madrid, Spain; ^6^ Department of Chemical Engineering and Food Technology, Science Faculty, University of Cádiz, Cádiz, Spain; ^7^ Instituto de Investigación Sanitaria Gregorio Marañón, Madrid, Spain; ^8^ High Performance Research Group in Physiopathology and Pharmacology of the Digestive System (NeuGut), Universidad Rey Juan Carlos, Madrid, Spain; ^9^ Departamento de Bioingeniería e Ingeniería Aeroespacial, Universidad Carlos III de Madrid, Madrid, Spain; ^10^ Centro Nacional de Investigaciones Cardiovasculares (CNIC), Madrid, Spain

**Keywords:** mangiferin, schizophrenia, Poly I:C, oxidative/nitrosative stress, neuroinflammation, magnetic resonace imaging (MRI)

## Abstract

There is evidence that in schizophrenia, imbalances in inflammatory and oxidative processes occur during pregnancy and in the early postnatal period, generating interest in the potential therapeutic efficacy of anti-inflammatory and antioxidant compounds. Mangiferin is a polyphenolic compound abundant in the leaves of *Mangifera indica L.* that has robust antioxidant and anti-inflammatory properties, making it a potential candidate for preventive or co-adjuvant therapy in schizophrenia. Hence, this study set-out to evaluate the effect of mango leaf extract (MLE) in a model of schizophrenia based on maternal immune activation, in which Poly I:C (4 mg/kg) is administered intravenously to pregnant rats. Young adult (postnatal day 60–70) or adolescent (postnatal day 35–49) male offspring received MLE (50 mg/kg of mangiferin) daily, and the effects of MLE in adolescence were compared to those of risperidone, assessing behavior, brain magnetic resonance imaging (MRI), and oxidative/inflammatory and antioxidant mediators in the adult offspring. MLE treatment in adulthood reversed the deficit in prepulse inhibition (PPI) but it failed to attenuate the sensitivity to amphetamine and the deficit in novel object recognition (NOR) induced. By contrast, adolescent MLE treatment prevented the sensorimotor gating deficit in the PPI test, producing an effect similar to that of risperidone. This MLE treatment also produced a reduction in grooming behavior, but it had no effect on anxiety or novel object recognition memory. MRI studies revealed that adolescent MLE administration partially counteracted the cortical shrinkage, and cerebellum and ventricle enlargement. In addition, MLE administration in adolescence reduced iNOS mediated inflammatory activation and it promoted the expression of biomarkers of compensatory antioxidant activity in the prefrontal cortex and hippocampus, as witnessed through the reduction of Keap1 and the accumulation of NRF2 and HO1. Together, these findings suggest that MLE might be an alternative therapeutic or preventive add-on strategy to improve the clinical expression of schizophrenia in adulthood, while also modifying the time course of this disease at earlier stages in populations at high-risk.

## Introduction

Schizophrenia (SZ) is a severe, chronic psychiatric disorder that affects around 24 million people worldwide (GBD 2019 Diseases and Injuries Collaborators, 2020). It is characterized by a set of heterogeneous symptoms that lead to impaired cognitive, social and occupational performance (rev. in Owen et al., 2016), and it is considered one of the leading causes of disability worldwide (GBD 2019 Mental Disorders Collaborators, 2022). Moreover, in addition to the deterioration in quality of life, patients with SZ experience a reduction in life expectancy of about 15 years (rev. in Hjorthøj et al., 2017). Therefore, even though SZ does not have a high prevalence (<1%), its huge impact on patients, their family and society makes it one of the most burdensome illnesses (Chong et al., 2016; Charlson et al., 2018).

Although the aetiology and biological substrates involved in SZ are still not fully understood, it is currently considered a neurodevelopmental disorder triggered by the interaction of a large number of genetic and/or environmental factors that alter normal brain development (rev. in Owen et al., 2016; McCutcheon et al., 2019). Although one of the most relevant risk factors is exposure to stressful events during the perinatal period (Van Os et al., 2010; Millan et al., 2016; Arango et al., 2021), epidemiological studies indicate that exposure to infections during pregnancy increases the risk of developing SZ in adulthood (rev. in Brown and Derkits, 2010). Accordingly, preclinical studies suggest that maternal immune activation is one of the key components in the post-pubertal emergence of SZ-like behavioral and neurobiological alterations (rev. in Gilmore and Jarskog, 1997; Talukdar et al., 2020). Likewise, pharmacological interventions during peri-adolescence can prevent the emergence of the behavioral and brain structural abnormalities produced by a prenatal insult (Zuckerman et al., 2003; Piontkewitz et al., 2009; Casquero-Veiga et al., 2021), while treatment in adulthood might only alleviate the symptomatology presented.

A large number of preclinical and clinical studies suggest that altered immune responses (e.g., increased brain-blood barrier permeability and glial activation), and changes to inflammatory pathways (cytokine imbalances) and in oxidative stress (redox dysregulation) are key aspects of the pathophysiology of SZ (rev. in Leza et al., 2015; Müller, 2018; Upthegrove and Khandaker, 2020). In addition, antioxidant and anti-inflammatory agents have been proposed as alternative or add-on therapeutic strategies to combat SZ. In fact, several studies showed beneficial effects of compounds with these pharmacological actions, such as N-acetylcysteine (Berk et al., 2008), α-lipoic acid (rev. in Salehi et al., 2019), vitamins C (Dakhale et al., 2005) and E (Soares-Weiser et al., 2018), minocycline (Romero-Miguel et al., 2021) or even omega-3 fatty acids (Casquero-Veiga et al., 2021).

Mangiferin (1,3,6,7-tetrahydroxyxanthone-C2-β-D-glucoside) is a bioactive polyphenolic molecule predominantly found in the leaves, bark, fruit skin and root of the mango tree (*Mangifera indica L.*). This compound has generated growing interest due to its anti-inflammatory and antioxidant properties but also, due to other biological activities that include anti-diabetic, analgesic, anti-microbial, anti-tumour or immunomodulatory effects (rev. in Saha et al., 2016; Du et al., 2018). These pharmacological activities have shown to improve inflammation and oxidative damage induced by stress (Márquez et al., 2012); anxiety- and depressive-like behaviors (Jangra et al., 2014) and cognitive deficit in a model of Alzheimer’s disease (Infante-Garcia et al., 2017b), suggesting a neuroprotective role of mangiferin. Furthermore, mangiferin improved the behavioral and oxidative damage produced by a ketamine model of SZ (Rao et al., 2012).

Therefore, in this study the aim was to evaluate the effect of administering a mango leaf extract (MLE) with a high content of mangiferin, in a model of SZ induced by maternal Poly I:C immune activation. This animal model is a well-establish model of SZ based on neurodevelopmental changes that mimics the clinical course of this disorder and the behavioral and neurobiological alterations observed in SZ patients (rev. in Meyer and Feldon, 2012). In this study, both young adult and adolescent rats were administered MLE, the effects of which were assessed through behavioral evaluations, magnetic resonance imaging (MRI) and biochemical measurement of inflammatory and antioxidant markers. As a result, we hoped to gain support for the testing and use of this extract to manage SZ.

## Materials and methods

### Animals

Male Wistar rats from the University of Cadiz were used in the experiments, maintained on a 12 h light/dark cycle with *ad libitum* access to food and water. All procedures were carried out in accordance with the European Communities Council Directive 2010/63/UE and they were approved by the Ethics Committee for Animal Experimentation at the School of Medicine of the University of Cadiz.

### Prenatal Poly I:C treatment

Prenatal Poly I:C treatment was performed as described previously (Zuckerman et al., 2003; Zuckerman and Weiner, 2003; Casquero-Veiga et al., 2019). On gestational day (GD) 15, pregnant dams were anesthetized with 4%–2% isoflurane and a single intravenous injection of the synthetic Poly I:C analogue of double-stranded RNA (4 mg/kg dissolved in saline; batch#37M4011V, Sigma Aldrich, Spain) was administered to their tail vein. An equivalent volume of saline alone (vehicle, VH) was injected into the control animals. Dams were weighed 0, 8, 24 and 48 h after Poly I:C or saline administration, and the body weight of their offspring was also evaluated on PND 1 (Lins et al., 2018; Supplementary Figure S1). On postnatal day (PND) 21, male pups were weaned and housed in groups of 2-4 per cage.

### Mango leaf extract (MLE)

Supercritical fluid extraction of mango leaves was used to obtain extracts with a high phenolic content and potent antioxidant activity. Briefly, mango (*Mangifera indica L.*) leaves were extracted by a high-pressure technique in a pilot-plant scale apparatus (model SF 2000; Thar Technology, PA, United States). Subcritical water was used as the extraction solvent at a pressure of 20 MPa and a temperature of 80°C based on our previous work (Fernández-Ponce et al., 2015). The use of solvents at high temperature and high pressure enhances the extraction performance as compared with conventional processes carried out at room temperature and atmospheric pressure (Fernández-Ponce et al., 2015; Casas-Cardoso et al., 2021). Three extraction procedures were carried out in batch mode for 12 h and subsequently, extracts were collected, sterilized and stored at −20°C in the absence of light until they were analyzed (Fernández-Ponce et al., 2015). The extract was characterized in terms of global extraction yield, antioxidant and anti-inflammatory activity, and the quantity of phenolic compounds. For each extraction procedure, the global extraction yield obtained was calculated as the ratio of the dry extract to the dry raw material and they were expressed as g extract/100 g raw material.

The antioxidant activity of the MLE was determined by means of the 2,2-diphenyl-1-picrylhydrazyl (DPPH) assay (Brand-Williams et al., 1995) and expressed as the antioxidant activity index (AAI): poor antioxidant activity when the AAI<0.5; moderate antioxidant activity when the AAI is 0.5–1.0; strong antioxidant activity when the AAI is 1.0–2.0; and very strong at an AAI >2.0 (Scherer and Godoy, 2009). Only a MLE obtained with a very strong antioxidant activity was used in this study. To determine the anti-inflammatory activity of the MLE, the capacity of the agent to prevent the denaturation of egg albumin was measured by spectrophotometry at 660 nm (Rahman et al., 2015), expressed as the concentration of 50% efficiency (IC_50_). The phenolic compounds in the extract were quantified by Ultra High-Performance Liquid Chromatography (UHPLC), as described previously (Fernández-Ponce et al., 2015) and using a Thermo Scientific Dionex Ultimate 3000 model with a Diode Array detector connected to the Chromeleon TM 7 software application for data analysis.

### Experimental design, groups and MLE treatment

 To study the effectiveness of MLE in reversing the alterations to Poly I:C offspring, two different treatment approaches were assessed, the therapeutic treatment in young adults and a preventive strategy treating during peri-adolescence.

**FIGURE 1:**
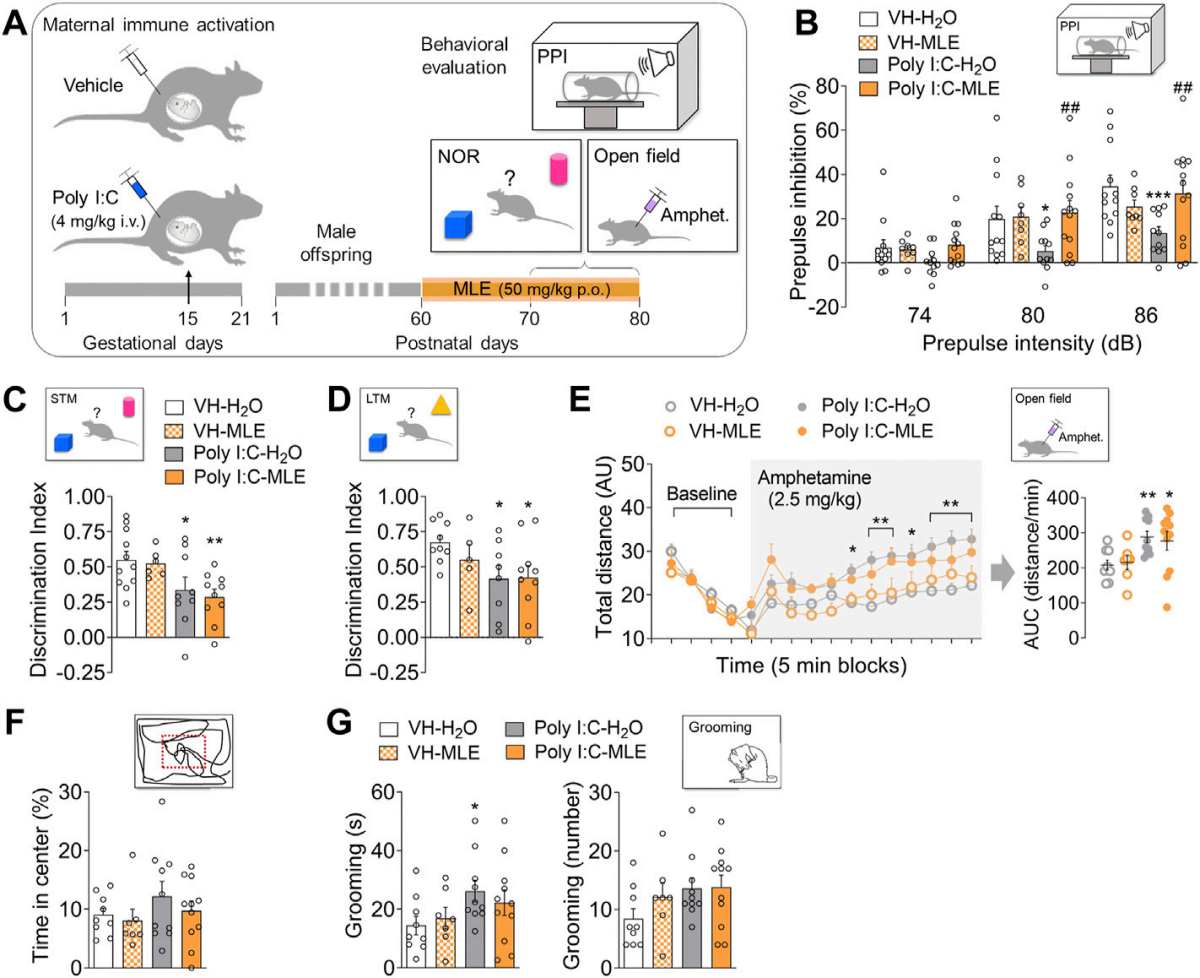
The effect of MLE treatment in adulthood on the behavior of the Poly I:C offspring. **(A)** Experimental timeline showing the design to study the effect of MLE treatment in adults. Maternal immune activation was induced in pregnant dams by administering Poly I:C (4 mg/kg i.v., intravenously) or the vehicle alone (controls) on gestational day (GD) 15. Male offspring were treated orally (p.o.) with MLE at a daily dose of 50 mg/kg of mangiferin in young adults since postnatal day (PND) 60. Then, behavioral evaluation was performed (PND 70-80). **(B)** In the prepulse inhibition (PPI) test, the effect of MLE treatment in adulthood is represented as the percentage PPI for 74, 80 and 86 dB prepulse intensity. **(C,D)** The effect of MLE treatment in adulthood on the object discrimination index for the short-term (STM) and long-term memory (LTM) phases of the novel object recognition test (NOR). **(E)** The effect of MLE treatment in adulthood on amphetamine sensitivity in the Poly I:C offspring. The total distance travelled in the open field is represented in 5 min (min) blocks, before and after amphetamine injection (shaded area, 2.5 mg/kg, i.p.). In addition, the area under the curve (AUC) values of amphetamine-induced activity were represented from 5 min after amphetamine injection. **(F)** The effect of MLE treatment in adulthood on anxiety-like behavior. The time spent (%) in the center is represented during the baseline period of free exploration in the open field. **(G)** The effect of MLE treatment in adulthood on grooming behavior. The total time spent grooming and number of grooming events are represented during the last 10 min of the baseline period in the open field. The data are represented as the mean ± SEM of 8–13 animals per group in the PPI test, *n* = 5-11 animals per group in STM and LTM phases of NOR test, and 9-11 animals per group in amphetamine-induced activity, anxiety-like and grooming behaviors. ^*^ *p* < 0.05, ^**^ *p* < 0.01, ^***^ *p* < 0.001 vs. VH-H_2_O; ^##^ *p* < 0.01 vs. Poly I:C-H_2_O, as assessed by two-way or two-way RM ANOVA followed by the LSD post-hoc test.

To assess the effect of MLE in young adults, four experimental groups were evaluated: male offspring derived from dams injected with Poly I:C or VH during pregnancy that were treated in adulthood with MLE (VH-MLE and Poly I:C-MLE groups) or drinking water (VH-H_2_O and Poly I:C-H_2_O groups). The MLE was orally administered and diluted in the drinking water based on their daily water consumption. Dosage was adjusted to 50 mg/kg of mangiferin per day on the basis of our experience and the previous data available in the literature using this phenolic compound (Márquez et al., 2012; Rao et al., 2012; Infante-Garcia et al., 2017a; Infante-Garcia et al., 2017b). To guarantee minimal phenolic degradation, MLE solutions was prepared freshly every day. In this treatment approach, the MLE was administered to young adults from PND 60 until the end of experiments and behavioral evaluation were carried out between PND 70-80 (Figure 1A).

**FIGURE 2:**
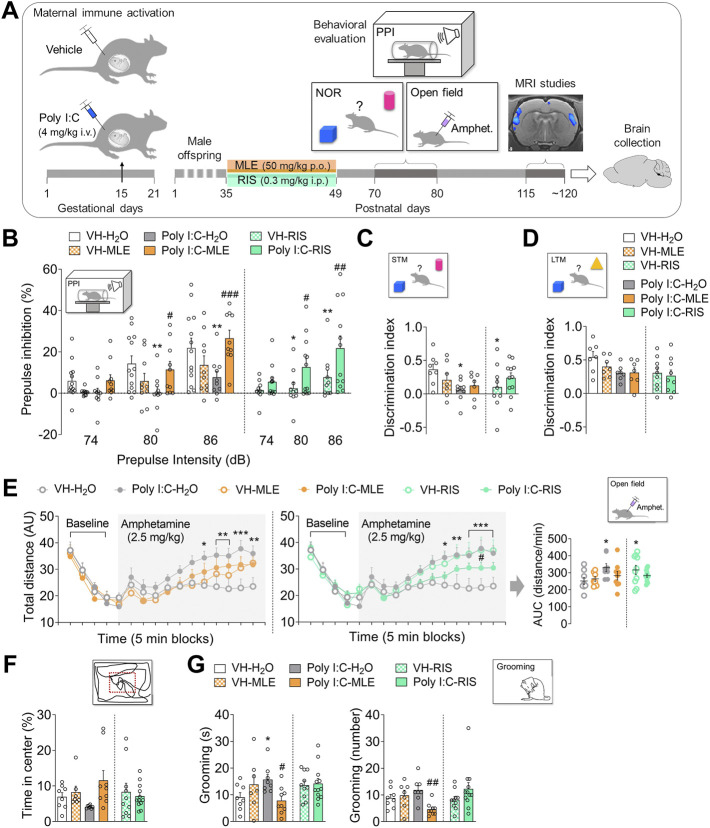
The effect of MLE or risperidone (RIS) treatment during adolescence on the behavior of the Poly I:C offspring. **(A)** Experimental timeline showing the design to study the effect of MLE treatment during adolescence. Maternal immune activation was induced in pregnant dams by administering Poly I:C (4 mg/kg i.v., intravenously) or the vehicle alone (controls) on gestational day (GD) 15. Male offspring were treated orally (p.o.) with MLE at a daily dose of 50 mg/kg of mangiferin during adolescence (postnatal day, PND 39-54) or administered intraperitoneally (i.p.) with risperidone (RIS, 0.3 mg/kg) as an adolescence reference treatment. Behavioral evaluation and magnetic resonance imaging (MRI) studies were performed at adulthood (PND 70-80 and PND 115-120, respectively). Finally, brain samples were collected at the end of the experiments. **(B)** The effect of adolescent MLE or RIS treatment on prepulse inhibition (PPI) test. The percentage PPI is represented for a 74, 80 and 86 dB prepulse intensity. **(C,D)** The effect of adolescent MLE or RIS treatment on the short-term (STM) and long-term memory (LTM) discrimination index between objects in the novel object recognition test (NOR). **(E)** The effect of adolescent MLE (left) or RIS (right) treatment on amphetamine sensitivity. The total distance travelled in the open field is represented in 5 min (min) blocks, before and after amphetamine injection (shaded area, 2.5 mg/kg i.p.). In addition, the area under the curve (AUC) values of amphetamine-induced activity were represented from 5 min after amphetamine injection. **(F)** The effect of adolescent MLE or RIS treatment on anxiety-like behavior. The time spent (%) in the center is represented during the baseline period of free exploration in the open field. **(G)** The effect of adolescent MLE or RIS treatment on grooming behavior. The total time spent grooming and the number of grooming events are represented during the last 10 min of the baseline period in the open field. The data are represented as the mean ± SEM of 10–13 animals per group in PPI test, 7-11 animals per group in the STM and LTM phases of the NOR test, and 7-12 animals per group for amphetamine induced activity, anxiety-like and grooming behaviors. ^*^ *p* < 0.05, ^**^ *p* < 0.01, ^***^ *p* < 0.001 vs. VH-H_2_O; ^#^ *p* < 0.05, ^##^ *p* < 0.01, ^###^ *p* < 0.001 vs. Poly I:C-H_2_O as assessed by two-way or two-way RM ANOVA followed by the LSD post-hoc test.

However, in order to evaluate the effect of MLE during the peri-adolescence period, male offspring derived from dams injected with Poly I:C or VH during pregnancy were treated during adolescence (PND 35-49) with MLE (VH-MLE and Poly I:C-MLE groups) or drinking water (VH-H_2_O and Poly I:C-H_2_O groups). MLE was also orally administered at the same dose of mangiferin (50 mg/kg) diluted in the drinking water. Preclinical and some clinical studies hint that the use of preventive treatments with atypical antipsychotics (e.g., risperidone) in patients at high risk of psychosis during the prodromal period of SZ could prevent the progression of the disease (Piontkewitz et al., 2011b; Marshall and Rathbone, 2011; Subotnik et al., 2015). In this way, the effects of risperidone during adolescence were evaluated. Thus, a set of offspring received a daily intraperitoneal administration (PND 35-49) of 0.3 mg/kg of risperidone (VH-RIS and Poly-RIS groups; Janssen Cork, Belgium). Behavioral evaluation was performed in adulthood (PND 70-80). Then, MRI studies were carried out (PND 115-120 approximately) and subsequently brain tissue was collected to evaluate the oxidative/inflammatory and antioxidant mediators (Figure 2A).

### Behavioral studies

Behavioral studies were performed on the adult offspring beginning at PND 70 and they included an assessment of prepulse inhibition of the startle response (PPI), novel object recognition (NOR), amphetamine-induced activity, anxiety-like and grooming behaviors*.*

#### Prepulse inhibition (PPI) test

PPI of the acoustic startle response was measured in a sound attenuated chamber using a movement-sensitive piezoelectric measuring platform (Cibertec, Spain). The test session began with a 10 min acclimatization to the startle chamber in the presence of 70 dB background noise, followed by five trials of startle stimulus (pulse, 120 dB). The rats were then subjected to 10 trials of pseudorandomly presented stimuli: pulse (120 dB); prepulse (74, 80 or 86 dB) + pulse (120 dB); or no stimulus (only background noise). Finally, they were subjected to five pulse trials (120 dB). The pulse and prepulse lasted 40 ms, the interval between the prepulse and pulse was set at 100 ms, whereas the inter-trial interval ranged from 10 to 20 s. The % PPI for each prepulse intensity was calculated from the pseudorandom presentations as follows: 100-[(startle response to prepulse + pulse)/startle response to pulse) x100)] (Casquero-Veiga et al., 2021).

#### Novel object recognition (NOR) test

Rats were tested in a plastic, black, square arena (45 × 45 × 35 cm) located in a room with dim lighting. The NOR memory tests were performed as described previously (Llorca-Torralba et al., 2019). Briefly, two identical objects were placed in the arena during the training phase and subsequently, NOR memory was evaluated in two test sessions 2–3 h (short-term memory, STM) and 24 h (long-term memory, LTM) after the training session: one with a familiar object and one with a novel object. Each session lasted 10 min. The objects used were of different shapes, colors and textures, and they were thoroughly cleaned with 70% ethanol between trials to ensure the absence of any olfactory cues. The time spent exploring each object was recorded and the relative exploration of the novel object was expressed as a discrimination index [DI = (t_novel_ - t_familiar_)/(t_novel_ + t_familiar_)](Llorca-Torralba et al., 2019). The criteria for exploration were based strictly on active exploration, and circling or sitting on the object were not considered exploratory behaviors.

#### Open field test

Locomotor activity was also measured in a plastic, black, square arena (45 × 45 × 35 cm) in dim lit boxes. Rats were placed individually in the arena for 20 min of free exploration and in this period, the baseline motor activity was evaluated, as were anxiety-like and grooming behaviors. The animals then received a single, intraperitoneal (i.p.) injection of amphetamine (2.5 mg/kg; Merck, France) and they were returned to the activity chamber for 60 min to evaluate their amphetamine-induced activity (modified from Yee et al., 2005). The sessions were videotaped and the total distance travelled was then analyzed using the SMART (Spontaneous Motor Activity Recording and Tracking) video 3.0 software (Panlab, Spain). Both spontaneous and amphetamine induced-activity were expressed in arbitrary units (AU), in 5 min blocks, and the area under the curve (AUC) values of the total distance travelled was represented from 5 min after amphetamine administration. The time spent in the central area of the arena (%) was measured in this 20 min free exploration phase, considered as anxiety-like behavior. For grooming behavior, the total time spent grooming and the number of grooming events were evaluated over the last 10 min of the free exploration period, when the explorative behavior was reduced.

### MRI studies

Anesthetized animals (sevoflurane, inhaled; Romero-Miguel et al., 2021) treated during adolescence were scanned (∼ PND 115-120) using a 7-T Biospec 70/20 scanner (Bruker, Germany). A coronal T2-weighted spin-echo sequence was acquired: TE = 33 ms, TR = 3732 ms, averages 2 and slice thickness 0.4 mm. The matrix size was 256 × 256 pixels at a FOV of 3.5 × 3.5 cm^2^.

Data processing and analysis. Two types of analysis were performed: a predefined regions of interest (ROI) analysis of primarily subcortical areas; and a voxel-based morphometry (VBM) approach of the entire brain. For the VBM analysis, MRI processing was performed as reported previously (Casquero-Veiga et al., 2019). T2 images were preprocessed, realigned and resliced to a rat brain template space using SPM12 software (Valdés-Hernández et al., 2011; http://www.fil.ion.ucl.ac.uk/spm/software/spm12). These data were used to create a custom brain template (Avants and Gee, 2004) and all the resliced images were registered to the template. The modulated images for the gray matter (GM), white matter (WM) and cerebrospinal fluid (CSF) were then obtained using the probabilistic maps of the brain template (Valdés-Hernández et al., 2011) and the Jacobian determinants from the spatial normalization process. Modulated images were smoothed with a 10 mm FWHM Gaussian filter and then used for statistical analyses with SPM12. A 1500 voxel clustering (spatial extent) threshold was applied to minimize type I error.

To analyze the ROIs, raw T2-images were registered to a common CT reference using the algorithms described in Gasull-Camós et al. (2017). Subsequently, five ROIs were manually segmented onto each MRI image in the whole brain, hippocampus, frontal lobe, all ventricles and fourth ventricle, according to the Paxinos and Watson Rat Brain Atlas (Paxinos and Watson, 2006).

### Oxidative/inflammatory and antioxidant evaluations

At the end of the experiments, animals treated during adolescence were anesthetized and their brains were rapidly dissected and frozen at −80°C. For biochemical studies, cytosolic fraction and nuclear extracts from the prefrontal cortex (PFC) and hippocampus tissue samples were prepared according to published protocols (MacDowell et al., 2013).

Western Blot: Inflammatory mediators as the inducible isoforms of nitric oxide synthase (iNOS) and cyclooxygenase (COX2), p38 MAP kinase (p38), lipid peroxidation product 4-hydroxynonenal (4-HNE) and the antioxidant pathway kelch-like ECH-associated protein 1 (Keap1), heme-oxygenase 1 (HO1), NAD(P)H:quinone oxidoreductase 1 (NQO1) were performed in cytosolic extracts (MacDowell et al., 2016). Protein levels were measured using the Bradford method, loaded onto electrophoresis gel and blotted onto a membrane using a semi-dry transfer system. The membranes were blocked with 5% BSA for 1 h at room temperature and probed overnight at 4°C with: rabbit anti-iNOS (sc-650, 1:750, BSA 2%; SCBT, Germany), goat anti-COX2 (sc-1747, 1:750, BSA 2.5%; SCBT, Germany), rabbit anti-phospho-p38 (sc-17852, 1:750 BSA 1%; SCBT, Germany), mouse anti-p38 (sc-7972, 1:750 BSA 1%; SCBT, Germany), mouse anti-4-HNE (MAB3249, 1:1,000 TBSt; R&D, United Kingdom), mouse anti-Keap1 (MAB3024, 1:1,000, TBSt; R&D, United Kingdom), mouse anti-4-HNE (MAB3249, 1:1,000, TBSt; R&D, United Kingdom), rabbit anti-HO1 (ab68477, 1:1,000, TBSt; abcam, United Kingdom), goat anti-NQO1 (sc16464, 1:750, BSA 1%; SCBT, Germany) and mouse anti-β-actin (A5441, 1:10,000, TBSt; Merck, France). These primary antibodies were detected with horseradish peroxidase-linked secondary antibodies by incubating for 1.5 h at room temperature, the binding of which was detected with an Odyssey Fc System (LICOR^®^, Germany) and ChemiDoc (Biorad, United States). All measurements were obtained at least three times in separate assays and the results were expressed relative to the controls. Supplementary Figures S2,S3 show uncropped blots and 2 replicates of the bands for each biomarker assessed in the PFC and hippocampus, respectively.

Nuclear Factor erythroid-related 2 (NRF2) activity was measured in nuclear extracts using a commercial ELISA-based kit (600590; Cayman Chemical, United State). For antioxidant status and enzyme activity, tissue samples were sonicated in 400 μl PBS (pH 7) containing a protease inhibitor cocktail (Complete^®^; Merck, France) and the homogenates were then centrifuged at 10,000 g for 15 min at 4°C. Supernatants were used for determinations of the Superoxide Dismutase (SOD, K028-H1; Arbor Assay, United State), Catalase (CAT, K033-H1; Arbor Assay, United State), Glutathione Peroxidase (GPx, 703102; Cayman Chemical, United State), Glutathione (GSH, K006-H1; Arbor Assay, United State) and Total Antioxidant Capacity (TAOC) was measured with a commercial kit based on the ABTS method (E-BC-K219-M; Elabscience, United State). Nitrites levels were measured by using the Griess method (Green et al., 1982). Briefly, in an acidic solution with 1% sulphanilamide and 0.1% NEDA, nitrites convert into a pink compound that is photometrically calculated at 540 nm in a microplate reader (Synergy 2; BioTek, United State).

### Statistical analysis

All the data are represented as the means ± S.E.M and the results were analyzed using STATISTICA 10.0 (StatSoft, United State), applying a Student’s t-test (unpaired, two-tailed), or a one-way or two-way analysis of variance (ANOVA), with or without repeated measures (RM), followed by a LSD post-hoc test. The differences were considered significant at *p* < 0.05 except for the VBM analysis where they were considered significant at *p* < 0.01 (uncorrected) (Supplementary Tables S1–S4).

## Results

### Prenatal Poly I:C treatment

Maternal immune activation was confirmed by the effect of administering Poly I:C or the vehicle alone (VH), on the body weight of both pregnant dams and their offspring. This indirect measurement was chosen to avoid extra maternal stress by collecting blood samples for inflammatory mediators’ evaluation. Thus, intravenous Poly I:C injection significantly reduced the dam’s weight relative to the VH dams when assessed 8 h after injection (*p* < 0.05; Supplementary Figure S1A). Furthermore, Poly I:C offspring also weighed less than VH pups on PND 1 (*p* < 0.01; Supplementary Figure S1B).

### MLE characterization

This extract was characterized in terms of its global extraction yield, its antioxidant and anti-inflammatory activities, as well as the presence of phenolic compounds (Table 1). Thus, subcritical water extraction at 20 MPa and 80°C gave a high global extraction yield (37.1 ± 0.6 g/100 g of dried leaves), producing an extract with an AAI value of 4.5 ± 0.4 μg DPPH/μg dried extract and an IC_50_ value of 104.5 ± 1.5 μg/ml. Thus, this extract had very strong antioxidant activity and substantial anti-inflammatory activity, in conjunction with a high total polyphenol content, which included major phenolic compounds like mangiferin, iriflophenones and gallic acid (Table 1).

**TABLE 1:** Mango leaf extract characteristics.

Global extraction yield (g/100 g dried leaves)	37.1 ± 0.6
Antioxidant activity (AAI, μg DPPH/μg dried extract)	4.5 ± 0.4
Anti-inflammatory activity (IC_50_, μg/mL)	104.5 ± 1.5
Phenolic compounds (g/100 g dried extract)	
mangiferin	11.11 ± 0.02
iriflophenone 3-C-β-d-glucoside	8.95 ± 1.03
iriflophenone 3-C-(2-O-p-hydroxybenzoyl)-β-d-glucoside	5.89 ± 0.50
gallic acid	2.84 ± 0.04
iriflophenone 3-C-(2-O-galloyl)-β-d-glucoside	2.14 ± 0.65
quercetin 3-d-galactoside	1.08 ± 0.08
quercetin 3-β-d-glucoside	0.42 ± 0.57

### MLE treatment of young adults

In these studies, the effects of administering MLE to young adults that were the offspring of dams that had received Poly I:C or VH during gestation were behaviorally examined (Figure 1A).

#### Behavioral studies

##### PPI test

The PPI test we performed to assess whether MLE treatment in young adults would be able to normalize the sensorimotor gating deficit described in the Poly I:C model. Thus, RM ANOVA tests revealed statistically significant differences in this test for the prepulse factor (*p* < 0.001), and a significant interaction between Poly I:C and MLE (*p* < 0.05), as well as between the prepulse, MLE and Poly I:C factors (*p* < 0.05; Supplementary Table S1). Note that the presentation of a prepulse inhibited the acoustic startle response to the pulse in control animals (VH-H_2_O) in an intensity-dependent manner (Figure 1B). A post-hoc test showed Poly I:C administration provoked a significant reduction in PPI relative to the controls (VH-H_2_O) for the 80 and 86 dB intensities (*p* < 0.05 and *p* < 0.001, respectively; Figure 1B). MLE prevented the reduction in PPI provoked by Poly I:C in the offspring relative to Poly I:C-H_2_O group both at 80 and 86 dB (*p* < 0.01; Figure 1B). However, MLE did not significantly modify the PPI values in VH animals (Figure 1B).

##### NOR test

The effect of MLE treatment in young adults on cognitive function were assessed in the NOR test. During this test, two different concepts were studied: the acquisition of information or learning and the storage of information or memory that were evaluated in the STM and LTM phases of this paradigm, respectively. In this sense, two-way ANOVA revealed Poly I:C produced significant differences in the discrimination index for both the STM (*p* < 0.01) and LTM (*p* < 0.05) phases of the paradigm, with no significant interaction between Poly I:C and MLE treatment (*p* > 0.05; Supplementary Table S1). Thus, there was a significant reduction in the STM discrimination index in Poly I:C animals (Poly I:C-H_2_O, *p* < 0.05; Poly I:C-MLE, *p* < 0.01), as well as in the LTM test session (both *p* < 0.05) relative to control animals (VH-H_2_O; Figures 1C,D). MLE treatment did not modify the discrimination index in either Poly I:C or VH offspring (*p* > 0.05; Figures 1C,D).

##### Amphetamine-induced activity

Locomotor activity in response to a systemic administration of amphetamine was measured in our model of SZ after MLE treatment in adulthood. The RM ANOVA highlighted the significant differences in total distance travelled following amphetamine administration (Poly I:C, *p* < 0.05; time, *p* < 0.001; time and Poly I:C interaction, *p* < 0.05; Supplementary Table S1). As such, a post-hoc test showed that amphetamine administration provoked an increase in motor activity over time in all the experimental groups, although this was significantly exacerbated in Poly I:C animals (min 50, *p* < 0.05; min 55–60, *p* < 0.01; min 65, *p* < 0.05; min 70–80, *p* < 0.01; Figure 1E). When the AUC values were analyzed, two-way ANOVA revealed a main effect of Poly I:C (*p* < 0.05; Supplementary Table S1). Thus, a locomotor hyperactivity was observed in Poly I:C offspring measured as a significant increase in the AUC relative to VH offspring (Poly I:C-H_2_O, *p* < 0.01; Poly I:C-MLE, *p* < 0.05; Figure 1E). However, MLE administration did not significantly affect the motor activity exhibited by Poly I:C or the VH animals (*p* > 0.05; Figure 1E).

##### Anxiety-like behavior

To evaluate anxiety-like behavior in this model of SZ and the effect of MLE administration in adulthood on this behavior, the time spent in the central area of the arena (%) was measured during the free exploration phase of the amphetamine-induced activity. However, no significant effects were found on the time spent in the central area of the arena in Poly I:C groups, irrespective of whether they received MLE or not (*p* > 0.05; Supplementary Table S1, Figure 1F).

##### Grooming behavior

Psychosis-like animal models often exhibit an excessive self-grooming phenotype (Kalueff et al., 2016). In this sense, to explore the effect of MLE treatment in young adults on this behavior, time spent and number of grooming events were measured in the Poly I:C-induced model of SZ. Thus, two-way ANOVA revealed significant differences in the total time spent grooming for Poly I:C factor (*p* < 0.05; Supplementary Table S1). The post-hoc test showed that Poly I:C-H_2_O animals spent more time grooming than the controls (VH-H_2_O, *p* < 0.05; Figure 1G). Nevertheless, the total grooming time was not modified by MLE administration in the offspring irrespective of whether the dams had received the VH alone or Poly I:C (*p* > 0.05; Figure 1G). Likewise, no significant effects were evident in the number of grooming events in the Poly I:C offspring even after MLE treatment (*p* > 0.05; Supplementary Table S1, Figure 1G).

### Adolescent MLE treatment

In these studies, the effects of administering MLE to peri-adolescent rats that were the offspring of dams that had received Poly I:C or VH during gestation were behaviorally examined. Then, MRI studies were conducted and finally, brains were collected to evaluate the oxidative/inflammatory and antioxidant mediators in the PFC and hippocampus (Figure 2A).

#### Behavioral studies

##### PPI test

The effect of adolescent MLE treatment on the sensorimotor gating response was assessed in adult Poly I:C offspring. Thus, the RM ANOVA revealed significant differences in the prepulse of the PPI test (*p* < 0.001), a significant interaction between Poly I:C and adolescent treatment (*p* < 0.01) and between the prepulse, treatment and Poly I:C (*p* < 0.05; Supplementary Table S2). The post-hoc test showed that MLE administration prevented the reduction in PPI showed in Poly I:C-H_2_O group at 80 and 86 dB (*p* < 0.05, *p* < 0.001, respectively; Figure 2B). Nevertheless, MLE did not modify the PPI values in control animals. As expected, risperidone treatment also significantly influenced PPI, increasing the PPI in Poly I:C offspring (80 dB, *p* < 0.05; 86 dB, *p* < 0.01) relative to Poly I:C-H_2_O group (Figure 2B). However, risperidone also significantly decreased PPI in the control (VH) offspring at both 80 (*p* < 0.05) and 86 dB (*p* < 0.01; Figure 2B).

##### NOR test

The possible preventing effect of MLE treatment in adolescents on NOR deficit induced by this model of SZ was evaluated at adulthood in this test. Two-way ANOVA revealed a significant interaction between Poly I:C and treatment on the discrimination index in the STM (*p* < 0.05) but it was not significant in the LTM phase (*p* > 0.05; Supplementary Table S2). Thus, Poly I:C animals showed a significantly lower discrimination index in the STM than control animals (Poly I:C-H_2_O relative to VH-H_2_O animals, *p* < 0.05; Figures 2C,D). MLE did not significantly modify the discrimination index in either Poly I:C offspring or in the control (VH) rats (*p* > 0.05; Figures 2C,D). In this paradigm, risperidone treatment did not influence the discrimination index in the STM or LTM phases in Poly I:C animals (*p* > 0.05; Figures 2C,D), although it did significantly reduce the discrimination index over STM in VH offspring (VH-RIS compared to VH-H_2_O animals, *p* < 0.05; Figure 2C) without affecting the performance of LTM (*p* > 0.05; Figure 2D).

##### Amphetamine-induced activity

Locomotor activity after amphetamine administration in adult Poly I:C offspring was evaluated in order to investigate whether adolescent MLE treatment was able to reverse the enhanced sensitivity to these compounds at adulthood. The RM ANOVA showed significant differences in the total distance travelled following amphetamine administration (time, *p* < 0.001; interaction between Poly I:C and treatment, *p* < 0.05 and interaction between time, treatment and Poly I:C, *p* < 0.01; Supplementary Table S2). Amphetamine administration increased the motor activity over time in all the experimental groups, although this effect was significantly stronger in Poly I:C-H_2_O rats than in the control animals (VH-H_2_O, min 60, *p* < 0.05; min 65–70, *p* < 0.01; min 75, *p* < 0.001; min 80, *p* < 0.01; Figure 2E). Although MLE administration did not affect motor activity in this paradigm (*p* > 0.05; Figure 2E), risperidone treatment significantly increased the total distance travelled in VH offspring (VH-RIS compared to VH-H_2_O animals, min 60, *p* < 0.05; min 65, *p* < 0.01; min 70–80, *p* < 0.001; Figure 2E) and it did reduce the hyperactivity induced by Poly I:C (min 75, *p* < 0.05; Figure 2E). Furthermore, AUC ANOVA revealed a significant interaction on the motor activity induced (*p* < 0.05; Supplementary Table S2), with larger AUC values in Poly I:C-H_2_O relative to the VH-H_2_O rats (*p* < 0.05; Figure 2E). However, MLE administration did not alter the AUC in VH or Poly I:C offspring (*p* > 0.05; Figure 2E). By contrast, control animals treated with risperidone had significantly higher AUC values relative to VH-H_2_O animals (*p* < 0.05; Figure 2E), although no significant effect was observed in Poly I:C animals administered risperidone (*p* > 0.05; Figure 2E).

##### Anxiety-like behavior

Likewise, in order to assess the effect of adolescent MLE treatment on anxiety-like behavior in Poly I:C offspring, we evaluated in adults the time spent in the central area of the arena during the free exploration phase of the amphetamine-induced activity test. However, two-way ANOVA did not reveal a significant effect of Poly I:C or treatment on anxiety-like behavior (*p* > 0.05; Supplementary Table S2; Figure 2F).

##### Grooming behavior

In addition to test the effect of adolescent MLE treatment in the previous behavioral paradigms, this treatment was also assessed on grooming behavior exhibited by the adult Poly I:C offspring. In this way, the ANOVA revealed a significant interaction regarding both the total time spent grooming and the number of grooming events (both *p* < 0.05; Supplementary Table S2). Thus, the post-hoc test showed that Poly I:C-H_2_O animals spent significantly more time grooming than the control animals (VH-H_2_O, *p* < 0.05; Figure 2G) and that MLE administration impeded this increase in Poly I:C offspring (*p* < 0.05; Figure 2G) accompanied by a reduction of grooming events (*p* < 0.01; Figure 2G). By contrast, no significant differences in grooming behavior were evident in VH animals and in Poly I:C offspring that were administered risperidone (*p* > 0.05; Figure 2G).

#### MRI studies

MRI studies using this model of SZ have identified volumetric changes in some brain areas that emerges at adulthood as a consequence of the maternal immune activation (Piontkewitz et al., 2011b; Casquero-Veiga et al., 2019). Therefore, adult offspring were scanned to evaluate whether MLE treatment during the peri-adolescence period was able to prevent brain structural abnormalities induced by this model of SZ.

##### VBM analysis

**FIGURE 3:**
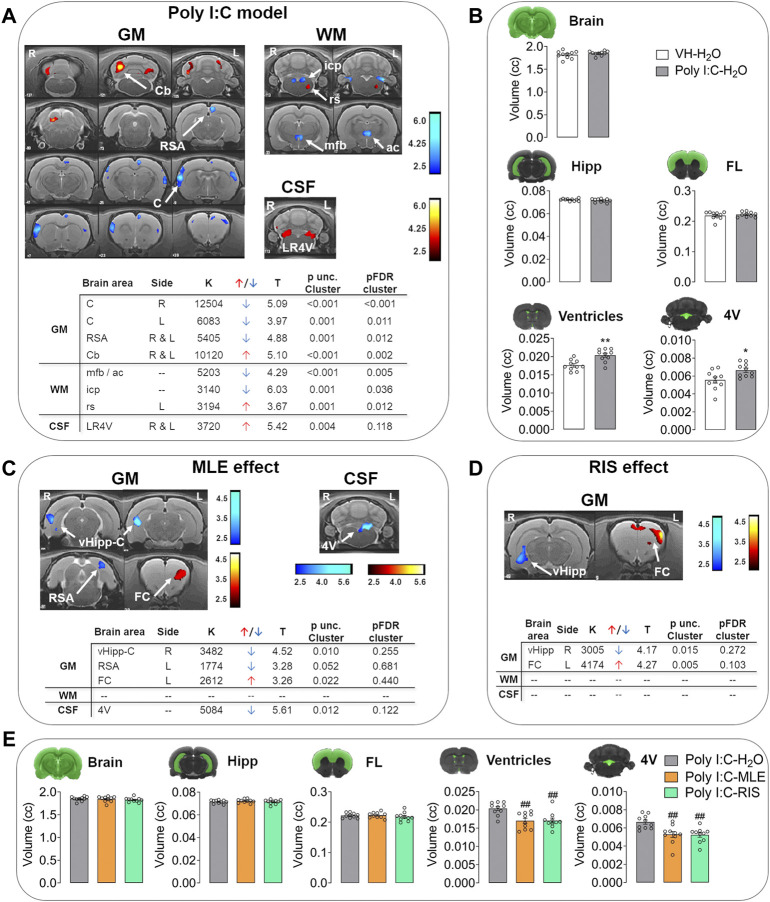
The effect of MLE or risperidone (RIS) treatment during adolescence on brain volumetric changes measured by MRI. **(A)** Voxel-based morphometry (VBM) and **(B)** regions of interest (ROI) analysis in the Poly I:C model (phenotype). VBM results are represented in T-maps overlaid on a T2-MR template showing the volumetric changes in the gray matter (GM), white matter (WM) and cerebrospinal fluid (CSF) in Poly I:C-H_2_O animals relative to the controls (VH-H_2_O). The color bars represent the T-values corresponding to volumetric enlargement (warm) and shrinkage (cold). Tables show the phenotype-related effects on brain volumetric changes in the GM, WM and CSF of Poly I:C animals from the VBM analysis. The ROI results are represented in column plots of global and regional volumetric changes in whole brain, hippocampus (Hipp), frontal lobe (FL), all ventricles and the fourth ventricle (4 V) of Poly I:C- H_2_O animals relative to the controls (VH-H_2_O). **(C)** VBM results after adolescent treatment with MLE or **(D)** RIS. The VBM results are represented in T-maps overlaid on a T2-MR template showing the volumetric changes in the GM, WM and CSF of Poly I:C offspring treated with MLE or RIS relative to Poly I:C-H_2_O animals. The color bars represent the T-values corresponding to volumetric enlargement (warm) and shrinkage (cold). The tables show treatment-related effects on the brain volumetric changes in the GM, WM and CSF of Poly I:C animals in the VBM analysis. **(E)** The ROI results after MLE or RIS treatment are represented as column plots of global and regional volumetric changes in whole brain, Hipp, FL, all ventricles and the 4 V compared to Poly I:C-H_2_O animals. The VBM tables include: side, right (R) and left (L); T, t value; k, cluster size; volume, increase (↑) or decrease (↓); p unc., *p* value uncorrected; FDR, false discovery rate. The data are represented as the mean ± SEM of 10 animals per group. ^*^ *p* < 0.05, ^**^ *p* < 0.01 vs. VH-H_2_O; ^##^ *p* < 0.01 vs. Poly I:C-H_2_O as assessed with a Student’s t-test (unpaired, two-tailed), or one-way ANOVA followed by the LSD post-hoc test. Abbreviations: AA, amygdaloid area; ac, anterior commissure; C, cortex; Cb, cerebellum; icp, inferior cerebellar peduncle; LR4V, lateral recess of the fourth ventricle; mfb, medial forebrain bundle; rs, rubrospinal tract; RSA, retrosplenial area; vHipp, ventral hippocampus.

VBM analysis revealed that Poly I:C animals showed a reduced GM volume in the frontal cortical areas and the retrosplenial cortex area, in addition to an enlargement of the GM in the cerebellum relative to the control animals (VH-H_2_O; Figure 3A). MLE treatment enlarged the GM in the frontal cortex in the Poly I:C animals, while it decreased it in the retrosplenial area and the ventral hippocampus-cortical area relative to Poly I:C-H_2_O rats (Figure 3C). In addition, there was a significant enlargement of the GM in the frontal cortex of Poly I:C animals treated with risperidone, coupled to a loss of GM in the ventral hippocampus relative Poly I:C-H_2_O animals (Figure 3D). Poly I:C immune activation also provoked a loss of WM volume in the inferior cerebellar peduncle, medial forebrain bundle and anterior commissure, along with WM enlargement in the rubrospinal tract compared to VH-H_2_O rats (Figure 3A). No significant changes in WM were found in Poly I:C animals that were then treated with MLE or risperidone (Figures 3C,D).

Poly I:C-H_2_O group showed CSF enlargements in the fourth ventricle relative to the control animals (VH-H_2_O; Figure 3A). However, MLE but not risperidone treatment significantly reduced this increase in the Poly I:C offspring (Figures 3C,D).

##### Manual ROI analysis

In evaluating the changes on ROIs analysis induced by Poly I:C animal model, a *t*-test showed significant increase were provoked in ventricular volume (*p* < 0.01), including the fourth ventricle (*p* < 0.05; Supplementary Table S3, Figure 3B). In terms of the effect of MLE and risperidone administration to offspring from dams that received Poly I:C, an ANOVA analysis showed a significant effect in ventricular brain regions (ventricles and fourth ventricle, *p* < 0.01; Supplementary Table S3), and the post-hoc test revealed that both MLE and risperidone prevented an enlargement of the ventricles (ventricles and fourth ventricle, *p* < 0.01; Figure 3E).

#### Oxidative/inflammatory and antioxidant mediators

**FIGURE 4:**
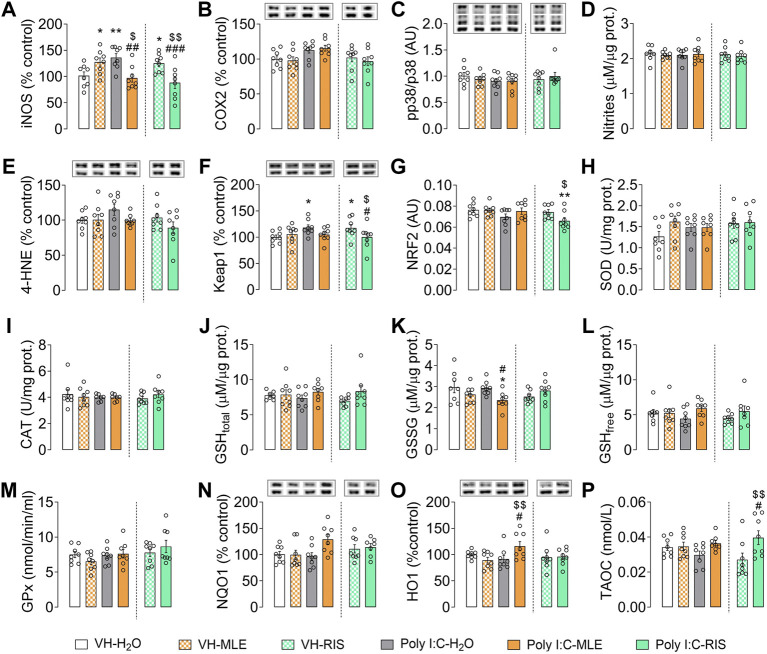
The effect of adolescent MLE or risperidone (RIS) treatment on the expression of oxidative/inflammatory mediators in the PFC. **(A–C)** The expression of the inflammatory mediators iNOS, COX2 and p38 relative to the controls, represented as the percentage (%) change, except for p38 which is represented as the ratio of phosphorylated relative to total p38 protein. **(D,E)** The concentrations of the indicators of oxidative/nitrosative damage (nitrites and 4-HNE) are expressed as µM/µg of protein, and as the % of the control expression. **(F–P)** Evaluation of the biomarkers of compensatory antioxidant mechanisms (Keap1, NRF2, SOD, CAT, GSH/GSSG, GPx, NQO1, HO1, TAOC). Keap1, NQO1 and HO1 expression is represented as the % of the controls; NRF2 activity is expressed in arbitrary units (AU); SOD and CAT enzyme activities are expressed as U/mg of protein; the levels of total (GSH_total_), oxidized (GSSG) and reduced (GSH_free_) glutathione are expressed as μM/µg of protein; Glutathione Peroxidase (GPx) is expressed as nmol/min/ml; and the Total Antioxidant Capacity (TAOC) is expressed in nmol/L. The data are represented as the mean ± SEM of 7-8 PFC samples per group. Representative bands of iNOS, COX2, 4-HNE, Keap1, NQO1 and HO1 (upper bands), and of the β-actin loading control (lower bands), are shown above their corresponding bars in the graph. For p38 protein expression, phosphorylated and total p38 representative bands are shown relative to β-actin (lower bands). ^*^ *p* < 0.05, ^**^ *p* < 0.01 vs. VH-H_2_O; ^$^ *p* < 0.05, ^$$^followed by the LSD post-hoc test.

Finally, after MRI studies, brains were collected to study the effect of adolescent MLE treatment on modulation of oxidative/inflammatory and antioxidant mediators in both the PFC and hippocampus of the adult Poly I:C offspring. Thus, iNOS and COX2 were assessed as two pro-inflammatory cytokines often overexpressed in this experimental model. In the PFC, ANOVA revealed an interaction (*p* < 0.001; Supplementary Table S4) of iNOS and the post-hoc test showed a significant increase in the iNOS protein levels in the Poly I:C animals (*p* < 0.01; Figure 4A), an effect that was prevented by MLE or risperidone administration (*p* < 0.01 and *p* < 0.001, respectively; Figure 4A). In terms of COX2, no significant differences were found (*p* > 0.05; Supplementary Table S4; Figure 4B). p38 signaling was also evaluated to know if this intracellular pathway contributes to the inflammatory process as well as indicators of oxidative/nitrosative damage (nitrites and 4-HNE, a specific lipid peroxidation marker) due to this damage is often triggered as consequence of that inflammatory response. However, no differences were found in the ratio of the pro-inflammatory mediator MAPK p38, nor in the oxidative/nitrosative damage markers based on nitrite levels or 4-HNE, a derivative of lipid peroxidation (*p* > 0.05; Figures 4C–E). In this context, adolescent treatment with this antioxidant extract could modify compensatory antioxidant mechanisms, so these mechanisms were assessed in terms of Keap1, NRF2, SOD, CAT, GSH/GSSG, GPx, NQO1, HO1 and TAOC. Similarly, no significant changes were observed in the PFC in some of the NRF2-dependent antioxidant compensatory mechanisms (SOD, CAT, GPx and NQO1; Figures 4H,I,M,N). However, ANOVA revealed an interaction with the Keap1 levels and with the antioxidant enzyme HO1 (both *p* < 0.05; Supplementary Table S4). In this sense, the post-hoc test revealed a significant increase in Keap1 levels in Poly I:C animals relative to the control group (VH-H_2_O, *p* < 0.05; Figure 4F) and an increased expression of HO1 after MLE administration to Poly I:C animals (Poly I:C-MLE compared to Poly I:C-H_2_O animals, *p* < 0.05; Figure 4O). A significant reduction in Keap1 levels was induced by risperidone treatment in Poly I:C animals (*p* < 0.05) whereas it increased these levels in control animals (VH-RIS, *p* < 0.05; Figure 4F). Regarding the nuclear NRF2 activity, a main effect of Poly I:C was found (*p* < 0.05; Supplementary Table S4) and the post-hoc test revealed that Poly I:C offspring treated with risperidone had lower levels of NRF2 than control groups (VH-H_2_O, *p* < 0.01; VH-RIS, *p* < 0.05; Figure 4G).

Similarly, the levels of total (GSH_total_), oxidized (GSSG) and reduced (GSH_free_) GSH were studied (Figures 4J–L), and a significant effect of treatment on GSSG was evident (*p* < 0.05), the post-hoc test revealing a significant decrease of GSSG in Poly I:C animals treated with MLE relative to the Poly I:C-H_2_O and VH-H_2_O rats (both *p* < 0.05; Figure 4K). Finally, two-way ANOVA revealed a significant interaction of the total antioxidant capacity of the samples (*p* < 0.05; Supplementary Table S4) and post-hoc test showed that risperidone treatment in Poly I:C rats increased this capacity relative to VH-RIS and Poly I:C-H_2_O groups (*p* < 0.01 and *p* < 0.05, respectively; Figure 4P).

**FIGURE 5:**
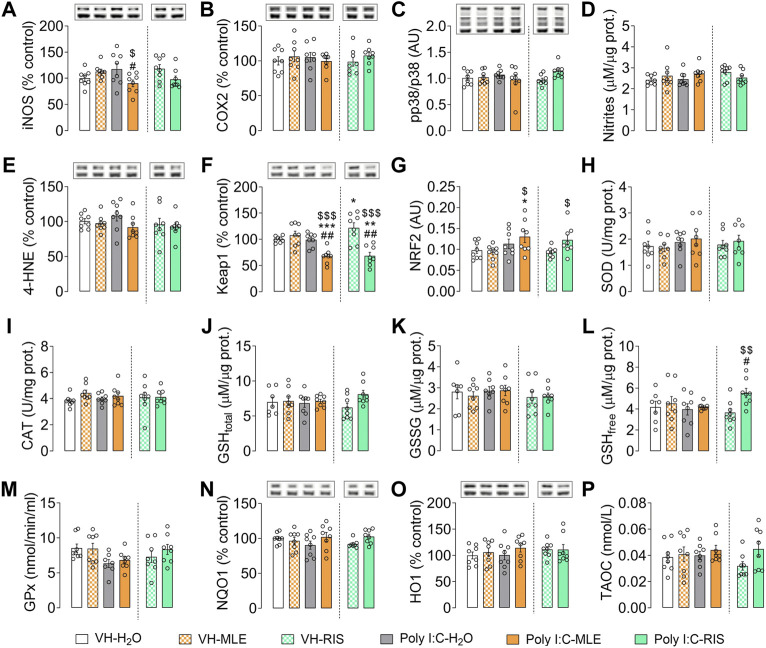
The effect of adolescent MLE or risperidone (RIS) treatment on the expression of oxidative/inflammatory mediators in the hippocampus. **(A–C)** The expression of the inflammatory mediators iNOS, COX2 and p38 represented as the percentage (%) of the control expression, with the exception of p38 which is represented as the ratio of phosphorylated relative to total p38 protein. **(D,E)** Indicators of oxidative/nitrosative damage (nitrites and 4-HNE) are expressed as their concentration (µM/µg of protein) and the % of the control expression, respectively. **(F–P)** The evaluation of biomarkers of compensatory antioxidant mechanisms (Keap1, NRF2, SOD, CAT, GSH/GSSG, GPx, NQO1, HO1, TAOC). Keap1, NQO1 and HO1 expression is represented as the % of the control expression; NRF2 activity is expressed in arbitrary units (AU); SOD and CAT enzyme activities are expressed as U/mg of protein; the levels of total (GSH_total_), oxidized (GSSG) and reduced (GSH_free_) glutathione are expressed as μM/µg of protein; Glutathione Peroxidase (GPx) is expressed in nmol/min/ml; and the Total Antioxidant Capacity (TAOC) is expressed in nmol/L. The data are represented as the mean ± SEM of 7-8 hippocampal samples per group. Representative bands of iNOS, COX2, 4-HNE, Keap1, NQO1 and HO1 (upper bands) and the β-actin loading control (lower bands) are shown above their corresponding bars in the graph. For p38 protein expression, phosphorylated and total p38 representative bands are shown relative to β-actin (lower bands). ^*^ *p* < 0.05, ^**^ *p* < 0.01, ^***^ *p* < 0.01 vs. VH-H_2_O; ^$^ *p* < 0.05, ^$$^ *p* < 0.01, ^$$$^ *p* < 0.001 vs. VH-MLE or VH-RIS; ^#^ *p* < 0.05, ^##^ *p* < 0.01 vs. Poly I:C-H_2_O as assessed by two-way ANOVA followed by the LSD post-hoc test.

In the hippocampus, the ANOVA analysis revealed an interaction for iNOS (*p* < 0.05; Supplementary Table S4), with the post-hoc tests demonstrating a significant decrease in the expression of iNOS in the Poly I:C offspring treated with MLE relative to the untreated animals (Poly I:C-H_2_O, *p* < 0.05; Figure 5A). No alterations to other inflammatory mediators were found, like COX2 or the p38 ratio, nor to nitrite or 4-HNE levels (*p* > 0.05; Supplementary Table S4; Figures 5B–E). However, ANOVA revealed a main effect for Poly I:C on Keap1 (*p* < 0.001) and a significant interaction (*p* < 0.001; Supplementary Table S4). Indeed, the post-hoc test showed that MLE significantly decreased the Keap1 levels in Poly I:C animals relative to the other groups of animals (VH-H_2_O and VH-MLE, *p* < 0.001; Poly I:C-H_2_O, *p* < 0.01; Figure 5F). The same reduction was induced by risperidone in Poly I:C animals (VH-H_2_O and Poly I:C-H_2_O, *p* < 0.01; VH-RIS, *p* < 0.001) but in VH offspring it significantly increased Keap1 levels (VH-RIS, *p* < 0.05; Figure 5F). This reduction in Keap1, an NRF2-inhibitory protein, was translated into an increase in nuclear NRF2 activity relative to the VH offspring (*p* < 0.05; Figure 5G), and ANOVA showed a significant effect of Poly I:C for NRF2 (*p* < 0.01). Two-way ANOVA indicated no change in the other antioxidant components (Figures 5H–K,M–P) except for a significant interaction of the free GSH (*p* < 0.05; Supplementary Table S4). The post-hoc test showed that Poly I:C animals treated with risperidone had higher levels of GSH_free_ than Poly I:C-H_2_O (*p* < 0.05) and VH-RIS animals (*p* < 0.01; Figure 5L).

## Discussion

This study demonstrates that the administration of MLE during adolescence or in adulthood exerts a beneficial effect in the maternal immune activation model of SZ, improving behavioral deficits particularly when administered in adolescence. In addition, MRI images and oxidative/inflammatory and antioxidant biomarkers were evaluated after adolescent treatment, with MLE partially counteracting the morphometric brain alterations detected in the SZ model (cortical shrinkage, and cerebellar and ventricles enlargement), and dampening the inflammatory iNOS pathway while enhancing antioxidative mechanisms. This effect was achieved with a MLE characterized by a strong antioxidant and anti-inflammatory activity, in conjunction with a higher content of mangiferin and other polyphenolic compounds, as seen previously (Liu et al., 2021). Moreover, the extraction method was environmentally friendly as it was based on high pressure extraction techniques that allow us to more efficiently recover more phenolic compounds from mango leaves with stronger antioxidant activity than conventional methods (Guamán-Balcázar et al., 2017).

According to previous studies, adult offspring from Poly I:C immune activated dams exhibit a sensorimotor gating deficit, reduced PPI, representing a core symptom of this animal model of SZ (Hadar et al., 2015; Casquero-Veiga et al., 2021). Cognitive impairment has also been observed in learning and memory phases of the NOR test, along with exacerbated amphetamine-induced activity (Zuckerman et al., 2003; Ozawa et al., 2006). Enhanced grooming behavior was also seen in Poly I:C offspring as a repetitive behavior related to maternal immune activation models (Amodeo et al., 2019).

To evaluate the effectiveness of MLE in reversing the alterations to Poly I:C offspring, two different treatment approaches were assessed, the therapeutic treatment in young adults and a preventive strategy during peri-adolescence, considering a prodromal and a critical therapeutic window for this neuropsychiatric disorder (rev. in Meyer et al., 2011; Piontkewitz et al., 2011b; Sommer and Arango, 2017). MLE administration to young adults produced a robust effect in the PPI test, reversing the sensorimotor gating deficit characteristic of this model of SZ. This effect of MLE on PPI was similar to that reported after treatment with an antipsychotic drug, e.g., clozapine (Ribeiro et al., 2013). However, MLE did not affect amphetamine-induced activity, recognition memory or grooming behavior. Previous studies suggest that this symptomatology might already be established by the time the animals received this adult MLE treatment (Ribeiro et al., 2013; Mattei et al., 2017; Talukdar et al., 2020). Chronic mangiferin treatment at the same dose as that used here improved the grooming and stereotyped abnormalities produced by an acute model of SZ induced by ketamine administration, as well as restoring IL-6 levels and lipid peroxidation (Rao et al., 2012). On the other hand, peri-adolescent MLE treatment prevented the emergence of the adult sensorimotor gating deficit and interestingly, the magnitude of this effect was similar to that of preventive treatment with risperidone or other antipsychotic drugs, or even that of anti-inflammatory/antioxidant compounds like polyunsaturated fatty acids (Meyer et al., 2010; Piontkewitz et al., 2011a; Casquero-Veiga et al., 2019; Casquero-Veiga et al., 2021; Romero-Miguel et al., 2021). MLE administration during adolescence also reverses the increased grooming behavior exhibited in Poly I:C offspring. Nevertheless, MLE failed to modify the development of sensitivity to amphetamines and the cognitive alterations seen in Poly I:C model. Therefore, although MLE prevent the hallmark PPI deficit in this model of SZ, unfortunately not all behavioral abnormalities could be halted by MLE administration in adulthood or adolescence.

Regarding the morphometric brain changes in the Poly I:C model, significant cortical shrinkage (frontal and retrosplenial) and ventricle enlargement was observed in this study, consistent with the brain alterations described previously in this model and in patients with SZ (Mitelman et al., 2005; Piontkewitz et al., 2009; Haijma et al., 2013; Casquero-Veiga et al., 2019). In addition, an augmentation on cerebellar areas was also seen in the offspring of Poly I:C dams. Morphometric changes in the cerebellum are not consistently reported in the literature, and while most previous studies have described a shrinkage, some reports have shown an enlargement of this brain area in SZ (Shenton et al., 2001; Lee et al., 2007; Moberget et al., 2018). Moreover, a reduction in WM fibers was observed, including a reduced volume of the anterior commissure/medial forebrain bundle and inferior cerebellar peduncle, along with an increased volume of the rubrospinal tract. Most of these WM alterations are consistent with WM deficits described in patients with SZ (Kubicki et al., 2007; Koch et al., 2010). Preventive MLE therapy during adolescence partially counteracts the morphometric changes observed in the offspring of Poly I:C dams. Thus, MLE increased the volume of the frontal cortex, in addition to reducing the ventricular and retrosplenial area volume. Bearing in mind the effect of risperidone detected by MRI previously (Piontkewitz et al., 2011a; Casquero-Veiga et al., 2019), the ROI analysis indicates that risperidone appears to reverse most brain structural alterations described in this model of SZ. A similar effect to the MRI changes to those achieved by this antipsychotic drug was observed after MLE treatment. The reversion of the PPI deficit found with MLE in Poly I:C offspring suggests that the brain structures involved in PPI processing might be preserved by MLE treatment during adolescence as occurred in the frontal cortex. Thus, although the corticostriatal-pallido-pontine circuit is the main neuroanatomic substrate underlying PPI processing, there is evidence that the PFC plays an important role in its modulation (Swerdlow et al., 2001; Tapias-Espinosa et al., 2019). Indeed, alterations to PFC integrity, activity and volume have been related to a PPI deficit (Koch and Bubser, 1994; Japha and Koch, 1999; Schneider and Koch, 2005; Uehara et al., 2007; Tapias-Espinosa et al., 2019) and conversely, the sensorimotor gating deficit observed in neurodevelopmental models of SZ has been related to abnormalities in the PFC (Day-Wilson et al., 2006; Wischhof et al., 2015; Toriumi et al., 2016). Moreover, frontal cortex preservation by adolescent MLE treatment could be involved in reducing grooming behavior that has been associated with alterations in corticostriatal connectivity (Kalueff et al., 2016; Wu et al., 2019). On the other hand, limited action of MLE on NOR test might be related with the inability to produce a beneficial effect on the hippocampus and the perirhinal cortex (Squire et al., 2007).

The offspring of Poly I:C dams showed an inflammatory process mediated by iNOS in the PFC accompanied by an increased in Keap1 levels as oxidative biomarker. The activation of these inflammatory and oxidative pathways has been described previously in this animal model of SZ (Casquero-Veiga et al., 2019; Romero-Miguel et al., 2021). However, no alterations were observed in other oxidative and antioxidant biomarkers in the adult Poly I:C offspring, at least at this point of the clinical course. MLE treatment during adolescence exerted an anti-inflammatory effect through the dampening of the iNOS pathway in both the PFC and hippocampus, similar to the effects of the antipsychotic risperidone, and those of other anti-inflammatory agents like polyunsaturated fatty acids (Casquero-Veiga et al., 2019; Casquero-Veiga et al., 2021). Indeed, the anti-inflammatory effects of mangiferin were previously proposed to be driven by reducing iNOS and COX2 expression (Bhatia et al., 2008; Márquez et al., 2012). Furthermore, adolescent MLE treatment promoted the expression of biomarkers of compensatory antioxidant mechanisms in the PFC and hippocampus of Poly I:C animals. Thus, a reduction in cytosolic Keap1 and GSSG was seen in Poly I:C offspring treated with MLE, accompanied by enhanced expression of nuclear NRF2 and HO1. These results indicate that the antioxidant effect of MLE was mediated by the NRF2-ARE pathway, contributing to NRF2 translocation to the nucleus, and the ensuing expression of the antioxidant enzyme HO1 (van Muiswinkel and Kuiperij, 2005). It has been widely demonstrated that mangiferin enhances the expression of NRF2 and its nuclear translocation exerts a neuroprotective role against inflammatory and oxidative stress via NRF2-ARE signaling (rev. in Du et al., 2018; Liu et al., 2021). Thus, MLE could improve the inflammatory imbalance evident in this model of SZ and promote antioxidant effects. Risperidone treatment also reduced Keap1 expression in the PFC and hippocampus, albeit with weaker antioxidant activity *via* the NRF2-ARE pathway. However, risperidone also had an effect on the antioxidant capacity enhancing the TAOC in the PFC. Note that the changes in oxidative, inflammatory and antioxidant biomarkers observed in this study may be the final or last mediators of these pathways, suggesting that the inflammatory mechanisms and oxidative damage occurred early in the time course of this model of SZ, and that MLE partially resolved these events.

Although the mechanisms involved in the beneficial effect of MLE must be further elucidated, as has been argued above, the reversal of the PPI deficit could be mainly due to the recovery of frontal cortex volume in conjunction with the inflammatory and antioxidative mechanisms that enhance in this brain area in the Poly I:C offspring. Furthermore, there is evidence of a correlation between the neuroprotective role of mangiferin, and the promotion of anti-inflammatory and antioxidant events, mainly in cortical brain areas (Márquez et al., 2012; Jangra et al., 2014; Infante-Garcia et al., 2017a; rev. in Liu et al., 2021). Therefore, the frontal cortex could represent the target area where the anti-inflammatory and antioxidant effect of MLE might act and produce its benefits.

This study is subject to some limitations including that we have only evaluated the effects of MLE in male offspring. This is mainly due to the fact that the female response to Poly I:C prenatal insult is lower and more variable than those of male animals (Haida et al., 2019). However, further studies with females must be performed to shed light on possible sex differences in the beneficial effect of MLE. Additionally, to avoid extra maternal stress by collecting blood samples for inflammatory mediators’ evaluation, the maternal immune activation was evaluated through the reduction in body weight of pregnant mothers after the inflammatory insult, which is an indirect measurement previously associated to Poly I:C-induced immune activation (Piontkewitz et al., 2011b; Howland et al., 2012; Missault et al., 2014; Lins et al., 2018). However, it would also be interesting to evaluate the immune response induced by Poly I:C in dams by measuring cytokines levels. Moreover, the peri-adolescent MLE treatment (PND 35-49) exhibited a limited efficacy. This limitation could come from the MLE per se. Despite the analysis of some aspects of the antioxidant and anti-inflammatory activity of MLE, it is a natural and heterogeneous extract which mixture could not be fully characterized. Nonetheless, better results could be achieved through sustained MLE treatment from adolescence until adulthood in this model of SZ. In this sense, it would be interesting to study if longer treatment could increase MLE efficacy and, considering the results found in NRF2, to discern if adolescent MLE treatment acts during this critical period by increasing anti-inflammatory/antioxidant mechanisms or perhaps by promoting neuronal plasticity and synaptogenesis.

In summary, the results presented here indicate that MLE administration in adulthood could reverse the sensorimotor gating deficit that is a hallmark of SZ, whereas its administration during adolescence completely prevents this deficit, while improving grooming behavior. The behavioral improvement produced by adolescent MLE treatment was accompanied by an improvement to both morphological brain alterations, and to the inflammatory and redox imbalance in this maternal immune activation model. Hence, this study demonstrates the efficacy of MLE therapy, not only in adults but also during adolescence, when it seems to be able to disrupt the pathological progression of the SZ-related phenotype induced by a prenatal Poly I:C insult. Consequently, these results suggest that peri-adolescence represents a critical neurodevelopment window where early pharmacological interventions with anti-inflammatory/antioxidant compounds may be relevant in the course of this psychiatric disease.

## Data Availability

The raw data supporting the conclusion of this article will be made available by the authors, without undue reservation.
