# Supplementary material for: The effects of mango leaf extract during adolescence and adulthood in a rat model of schizophrenia
Source: Front Pharmacol. 2022 Jul 26;13:886514. doi: 10.3389/fphar.2022.886514 (PMC9360613; doi:10.3389/fphar.2022.886514)
Supplement: Supplementary file 1 [file DataSheet1.docx]

| **Supplementary Table S1. Summary of statistical analysis of Figure 1** | | | | | | | |
| --- | --- | --- | --- | --- | --- | --- | --- |
| **Two-way RM ANOVA** | **Poly I:C** | **MLE** | **Poly I:C*MLE** | **Prepulse** | **Poly I:C*Prepulse** | **MLE*Prepulse** | **Poly I:C*MLE*Prepulse** |
| **PPI test** | F_1,40_ = 1.88 | F_1,40_ = 2.17 | **F_1,40_ = 4.99*** | **F_2,80_ = 65.70***** | F_2,80_ = 1.18 | F_2,80_ = 1.54 | **F_2,80_ = 3.35*** |
|  |  |  |  |  |  |  |  |
|  |  |  |  | **Time** | **Poly I:C*Time** | **MLE*Time** | **Poly I:C*MLE*Time** |
| **Amphetamine-IA** | **F_1,33_ = 11.42**** | F_1,33_ = 0.01 | F_1,33_ = 0.18 | **F_11,363_ = 23.78***** | F_11,363_ = 0.78 | F_11,363_ = 0.95 | **F_11,363_ = 1.85*** |
|  |  |  |  |  |  |  |  |
| **Two-way ANOVA** |  |  |  |  |  |  |  |
| **AUC** | **F_1,33_ = 11.19**** | F_1,33_ = 0.93 | F_1,33_ = 0.67 |  |  |  |  |
| **Anxiety-like behavior** | F_1,33_ = 0.22 | F_1,33_ = 0.38 | F_1,33_ = 0.70 |  |  |  |  |
| **Grooming behavior** |  |  |  |  |  |  |  |
| Time spent (s) | **F_1,33_ = 4.67*** | F_1,33_ = 0.03 | F_1,33_ = 0.67 |  |  |  |  |
| Number of events | F_1,33_ = 2.90 | F_1,33_ = 0.95 | F_1,33_ = 0.75 |  |  |  |  |
| **NOR test** |  |  |  |  |  |  |  |
| STM | **F_1,32_ = 10.43**** | F_1,32_ = 0.30 | F_1,32_ = 0.03 |  |  |  |  |
| LTM | **F_1,28_ = 4.89*** | F_1,28_ = 0.44 | F_1,28_ = 0.59 |  |  |  |  |
| The F values were expressed with their associated degrees of freedom. PPI, prepulse inhibition; Poly I:C, polyriboinosinic-polyribocytidylic acid; MLE, mango leaf extract; amphetamine-IA, amphetamine induced activity; AUC, area under the curve; NOR, new object recognition; STM, short-term memory; LTM, long-term memory. *p<0.05; **p<0.01; ***p<0.001. | | | | | | | |

| **Supplementary Table S2. Summary of statistical analysis of Figure 2** | | | | | | | |
| --- | --- | --- | --- | --- | --- | --- | --- |
| **Two-way RM ANOVA** | **Poly I:C** | **Treatment** | **Poly I:C*Treatment** | **Prepulse** | **Poly I:C*Prepulse** | **Treatment*Prepulse** | **Poly I:C*Treatment*Prepulse** |
| **PPI test** | F_1,59_ = 0.65 | F_2,59_ = 0.32 | **F_2,59_ = 7.01**** | **F_2,118_ = 56.31***** | F_2,118_ = 1.21 | F_4,118_ = 1.03 | **F_4,118_ = 2.94*** |
|  |  |  |  |  |  |  |  |
|  | **Poly I:C** | **Treatment** | **Poly I:C*Treatment** | **Time** | **Poly I:C*Time** | **Treatment*Time** | **Poly I:C*Treatment*Time** |
| **Amphetamine-IA** | F_1,48_ = 1.21 | F_2,48_ = 0.95 | **F_2,48_ = 3.90*** | **F_11,528_= 31.03***** | F_11,528_ = 0.73 | F_22,528_ = 0.88 | **F_22,528_ = 2.05**** |
|  |  |  |  |  |  |  |  |
| **Two-way ANOVA** | **Poly I:C** | **Treatment** | **Poly I:C*Treatment** |  |  |  |  |
| **AUC** | F_1,48_ = 1.54 | F_2,48_ = 0.81 | **F_2,48_ = 3.72*** |  |  |  |  |
| **Anxiety-like behavior** | F_1,48_ = 0.02 | F_2,48_ = 2.59 | F_2,48_ = 1.37 |  |  |  |  |
| **Grooming behavior** |  |  |  |  |  |  |  |
| Time spent (s) | F_1,48_ = 0.05 | F_2,48_ = 1.12 | **F_2,48_ = 4.10*** |  |  |  |  |
| Number of events | F_1,48_ = 0.19 | F_2,48_ = 1.91 | **F_2,48_ = 4.08*** |  |  |  |  |
| **NOR test** |  |  |  |  |  |  |  |
| STM | F_1,45_ = 1.42 | F_2,45_ = 0.19 | **F_2,45_ = 3.70*** |  |  |  |  |
| LTM | F_1,40_ = 3.83 | F_2,40_ = 1.80 | F_2,40_ = 0.89 |  |  |  |  |
| The F values were expressed with their associated degrees of freedom. PPI, prepulse inhibition; Poly I:C, polyriboinosinic-polyribocytidylic acid; amphetamine-IA, amphetamine induced activity; AUC, area under the curve; NOR, new object recognition; STM, short-term memory; LTM, long-term memory. *p<0.05; **p<0.01; ***p<0.001. | | | | | | | |

| **Supplementary Table S3. Summary of statistical analysis of Figure 3** | | | | | |
| --- | --- | --- | --- | --- | --- |
|  | **Brain** | **FL** | **Hipp** | **Ventricles** | **4V** |
| **Unpaired Student’s t-test** | t_18_ = 1.10 | t_18_ = 0.25 | t_18_ = 1.08 | **t_18_ = 3.87**** | **t_18_ = 2.67*** |
| **One-way ANOVA** | F_2,27_ = 0.53 | F_2,27_ = 0.63 | F_2,27_ = 0.63 | **F_2,27_ = 8.74**** | **F_2,27_ = 8.63**** |
| The F values were expressed with their associated degrees of freedom. FL, frontal lobe; Hipp, hippocampus; 4V, fourth ventricle. *p<0.05; **p<0.01. | | | | | |

| **Supplementary Table S4. Summary of statistical analysis of Figures 4-5** | | | | |
| --- | --- | --- | --- | --- |
| **Parameter** | **Area** | **Poly I:C** | **Treatment** | **Poly I:C* Treatment** |
| **iNOS** | PFC | F_1,42_ = 2.67 | F_2,42_ = 1.09 | **F_2,42_ = 11.84***** |
|  | Hipp | F_1,42_ = 1.97 | F_2,42_ = 0.59 | **F_2,42_ = 4.76*** |
| **COX2** | PFC | F_1,42_ = 3.92 | F_2,42_ = 1.26 | F_2,42_ = 2.71 |
|  | Hipp | F_1,42_ = 0.24 | F_2,42_ = 0.00 | F_2,42_ = 0.89 |
| **pp38/p38 ratio** | PFC | F_1,42_ = 0.37 | F_2,42_ = 0.35 | F_2,42_ = 0.98 |
|  | Hipp | F_1,42_ = 1.82 | F_2,42_ = 0.58 | F_2,42_ = 1.91 |
| **Nitrites** | PFC | F_1,41_ = 0.12 | F_2,41_ = 0.10 | F_2,41_ = 0.22 |
|  | Hipp | F_1,42_ = 0.14 | F_2,42_ = 1.67 | F_2,42_ = 0.97 |
| **4-HNE** | PFC | F_1,42_ = 0.00 | F_2,42_ = 1.47 | F_2,42_ = 2.47 |
|  | Hipp | F_1,42_ = 0.00 | F_2,42_ = 1.71 | F_2,42_ = 0.65 |
| **Keap1** | PFC | F_1,42_ = 0.00 | F_2,42_ = 0.25 | **F_2,42_ = 4.57*** |
|  | Hipp | **F_1,42_ = 36.90***** | F_2,42_ = 1.79 | **F_2,42_ = 9.33***** |
| **NRF2** | PFC | **F_1,42_ = 6.01*** | F_2,42_ = 2.15 | F_2,42_ = 1.10 |
|  | Hipp | **F_1,42_ = 11.44**** | F_2,42_ = 0.21 | F_2,42_ = 0.64 |
| **SOD** | PFC | F_1,42_ = 0.16 | F_2,42_ = 1.95 | F_2,42_ = 1.13 |
|  | Hipp | F_1,42_ = 1.91 | F_2,42_ = 0.04 | F_2,42_ = 0.19 |
| **CAT** | PFC | F_1,42_ = 0.82 | F_2,42_ = 0.24 | F_2,42_ = 0.12 |
|  | Hipp | F_1,42_ = 0.01 | F_2,42_ = 1.31 | F_2,42_ = 0.30 |
| **GSH_total_** | PFC | F_1,42_ = 0.42 | F_2,42_ = 0.27 | F_2,42_ = 2.02 |
|  | Hipp | F_1,41_ = 1.42 | F_2,41_ = 0.10 | F_2,41_ = 2.05 |
| **GSSG** | PFC | F_1,42_ = 0.00 | **F_2,42_ = 4.00*** | F_2,42_ = 1.44 |
|  | Hipp | F_1,41_ = 0.35 | F_2,41_ = 0.65 | F_2,41_ = 0.14 |
| **GSH_free_** | PFC | F_1,42_ = 0.49 | F_2,42_ = 1.06 | F_2,42_ = 1.84 |
|  | Hipp | F_1,41_ = 1.27 | F_2,41_ = 0.57 | **F_2,41_ = 3.28*** |
| **GPx** | PFC | F_1,42_ = 1.68 | F_2,42_ = 2.04 | F_2,42_ = 0.65 |
|  | Hipp | F_1,42_ = 0.18 | F_2,42_ = 0.71 | F_2,42_ = 0.55 |
| **NQO1** | PFC | F_1,41_ = 2.31 | F_2,41_ = 2.59 | F_2,41_ = 2.59 |
|  | Hipp | F_1,41_ = 0.33 | F_2,41_ = 0.40 | F_2,41_ = 2.55 |
| **HO1** | PFC | F_1,42_ = 1.37 | F_2,42_ = 0.76 | **F_2,42_ = 3.64*** |
|  | Hipp | F_1,42_ = 0.21 | F_2,42_ = 1.15 | F_2,42_ = 0.19 |
| **TAOC** | PFC | F_1,42_ = 2.15 | F_2,42_ = 0.84 | **F_2,42_ = 4.83*** |
|  | Hipp | F_1,42_ = 2.71 | F_2,42_ = 0.50 | F_2,42_ = 1.04 |
| The F values were expressed with their associated degrees of freedom. Poly I:C, polyriboinosinic-polyribocytidylic acid; iNOS, inducible nitric oxide synthase; COX2, cyclooxygenase 2; pp38 phosphorylated-p38; 4-HNE, 4-hydroxynonenal; Keap1, kelch-like ECH-associated protein 1; NRF2, nuclear factor erythroid-related 2; SOD, superoxide dismutase; CAT, catalase; GSH_total_, glutathione total; GSSG, glutathione disulfide; GSH_free_, glutathione _free_; GPx, glutathione peroxidase; NQO1, NAD(P)H:quinone oxidoreductase 1; HO1, heme-oxygenase 1; TAOC, total antioxidant capacity. *p<0.05; **p<0.01; ***p<0.001. | | | | |


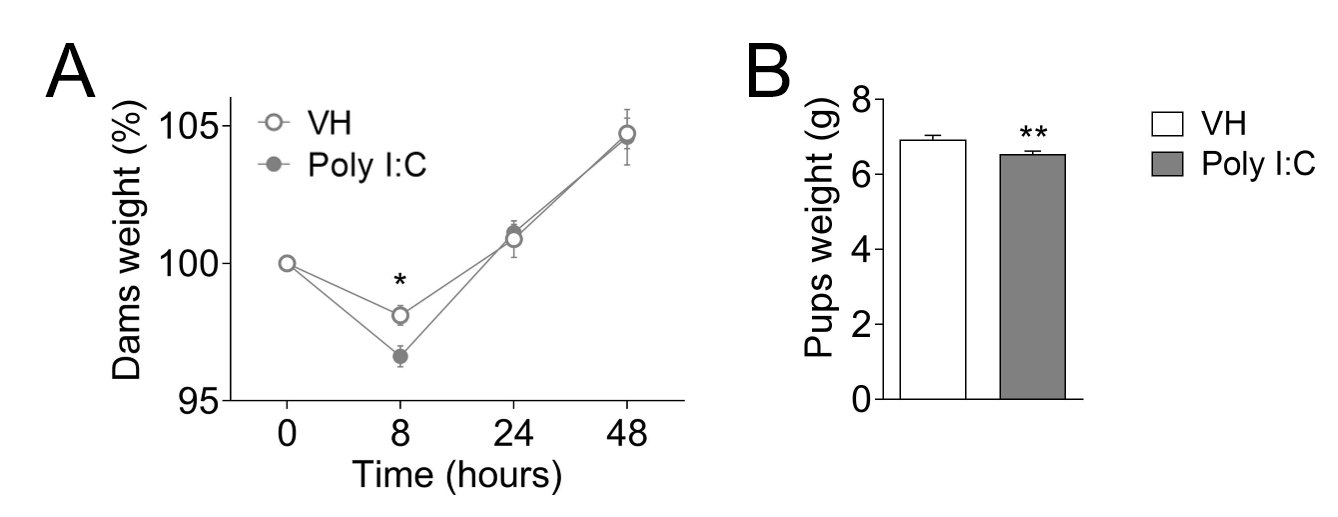


**Supplementary Figure S1. Maternal immune activation verification. A)** Effect of Poly I:C (4 mg/kg) and vehicle (VH, saline) intravenously administration on body weight in pregnant dams. Dams were weighted at 0, 8, 24 and 48 hours after Poly I:C or VH administration on gestational day (GD) 15. Body weight of pregnant dams is represented as percentage (%) of change from baseline. **B)** Effect of maternal immune activation on body weight of the offspring. Pups were weighted on postnatal day (PND) 1 and body weight is represented in grams (g). ^*^p<0.05, ^**^p<0.01 vs. VH as assessed by Student’s t-test (unpaired, two-tailed).

**
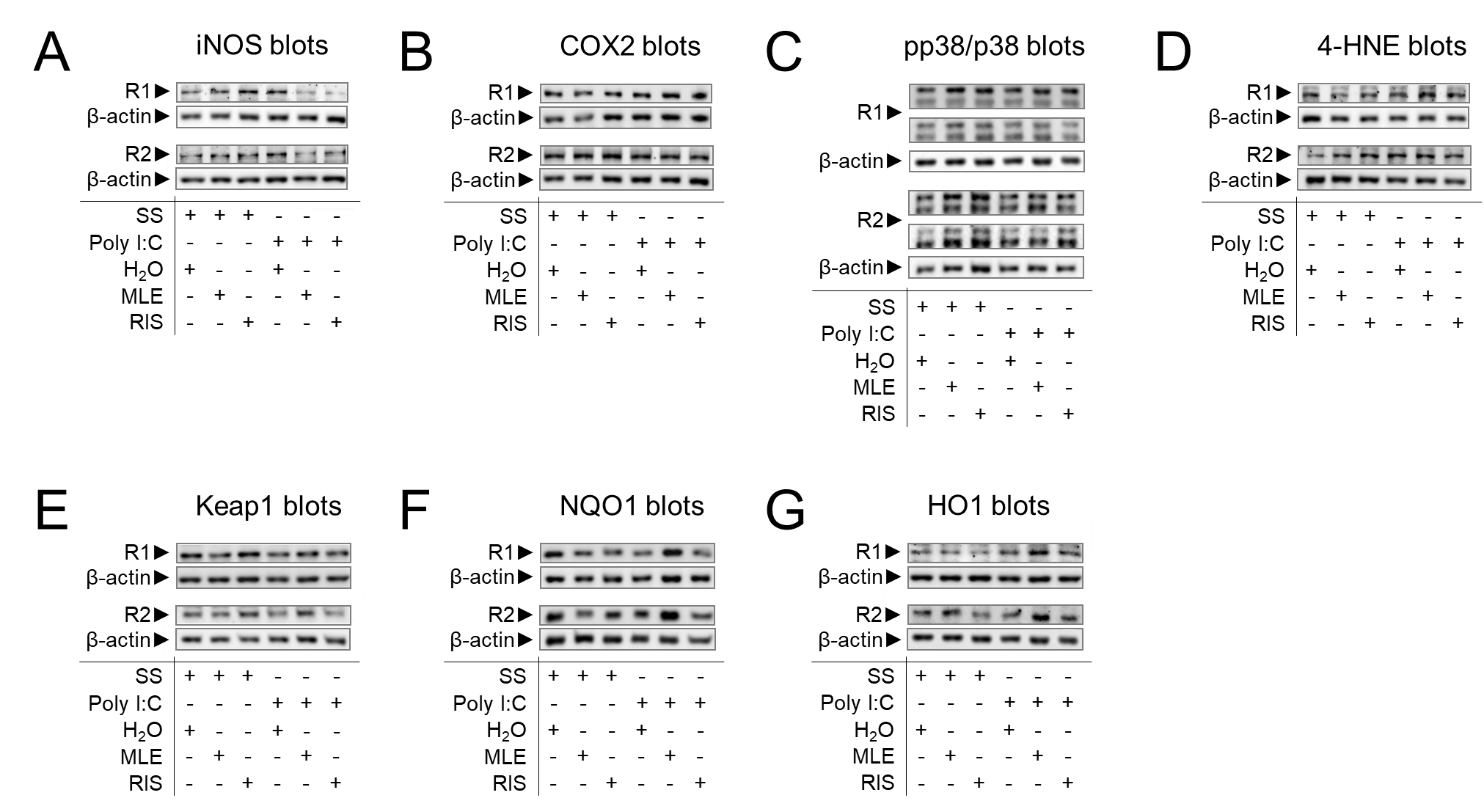
**

**Supplementary Figure S2. Blots for iNOS, COX2, p38, 4-HNE, Keap1, NQO1 and HO1 in the PFC.** Representative bands of 2 replicates (R1 and R2) of iNOS **(A)**, COX2 **(B)**, pp38/p38 **(C)**, 4-HNE **(D)**, Keap1 **(E)**, NQO1 **(F)** and HO1 **(G)** and of the β-actin loading control of the PFC of each experimental group (SS-H_2_O, SS-MLE, SS-RIS, Poly I:C-H_2_O, Poly I:C-MLE, Poly I:C-RIS).

**
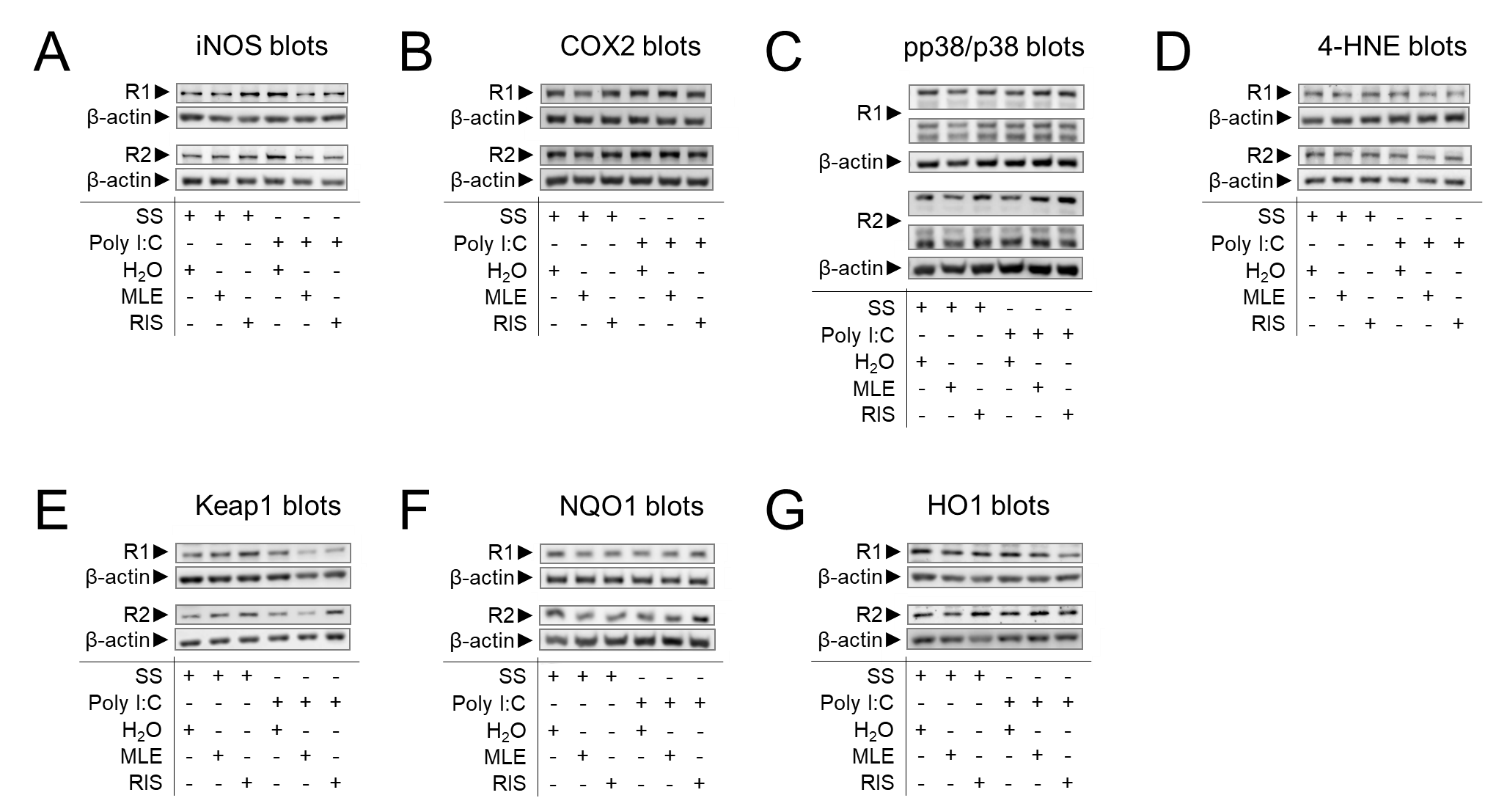
**

**Supplementary Figure S3. Blots for iNOS, COX2, p38, 4-HNE, Keap1, NQO1 and HO1 in the hippocampus.** Representative bands of 2 replicates (R1 and R2) of iNOS **(A)**, COX2 **(B)**, pp38/p38 **(C)**, 4-HNE **(D)**, Keap1 **(E)**, NQO1 **(F)** and HO1 **(G)** and of the β-actin loading control of the PFC of each experimental group (SS-H_2_O, SS-MLE, SS-RIS, Poly I:C-H_2_O, Poly I:C-MLE, Poly I:C-RIS).
